# Medical Portrait Gallery. Biographical Memoirs of the Most Celebrated Physicians, Surgeons, &c. Who Have Contributed to the Advancement of Medical Science

**Published:** 1838-07

**Authors:** 


Art. III.—*Medical Portrait Gallery. Biographical Memoirs of the most celebrated Physicians, Surgeons, &c. &c., who have contributed to the advancement of Medical Science.* By Thomas Joseph Pettigrew, F.R.S. &c. &c. &c.—*London and Paris,* 1838. 8vo. *In Monthly Parts; each Part containing three Portraits.* Parts I. II. and III.

Here is a most attractive publication, which nothing can mar but the manner of the execution. Of the portraits, generally, we may speak very favorably; but of their arrangement we can say little in praise: so incongruous, strange, and fantastic is that admired disorder, and so wonderful the contempt of concatenation as well as of chronology, which brings together, in Part I., Æsculapius, Albinus, and Sir Henry Halford; in Part II., Ruysch, Haller, and Sir Anthony Carlisle; and, in Part III., Linacre, Akenside, and Sir Charles Clarke. There is a most unfortunate degree of absurdity in these unions.

We greatly lament the want of a more useful, or, we might say, of a more independent plan, in what must otherwise become a very popular work. A history of medicine, in a series of biographical memoirs, and illustrated by portraits, would be interesting and instructive; as would also a work so arranged as to present the cultivators of different branches of medicine, with reference to some order or other: but a publication, like the present, which, instead of this, seems especially devoted to the gratification of living vanity, cannot possibly have all the success which Mr. Pettigrew's friends must desire. It may please the flattered to read their glowing epitaphs whilst yet alive; but the public, who should be the purchasers, are not sufficiently sentimental to pay for verse or prose so dedicated. It may gratify Sir Henry Halford to read that he has shed splendour around the College, and zealously laboured towards placing the members of the profession "in that station in society to which their merits, their education, and their utility justly entitle them;" but the members of that profession will not easily acquit Mr. Pettigrew of covert satire in these expressions, which have the misfortune to be so framed as to imply neglect on the one hand, thus delicately reproved, or demerit on the other, thus held up to the scorn of the politer physicians of London. We must confess that a perusal of a few passages of this kind has very much disinclined us to look steadily through the notices. The pen of history should be the pen of truth; but in this work the life of every living man becomes an inflated panegyric. As a collection of portraits, however, the work will still possess much interest. The newspaper critics are, we see, in ecstasies with the publication; but we sincerely hope Mr. Pettigrew will be rewarded by more discriminate praise. It is but just to him to quote a part of his address to the subscribers, accompanying the last of the Numbers published, and accounting for what seems disorderly arrangement. As the work is but commencing, we yet entertain a hope that, in its progress, we shall be able to speak of it more according to our desire. Perhaps, with the same good intention, we may venture to say a few words on the classical taste of the mottoes, prefixed, without any particular necessity, to each life. To the section headed Æsculapius is attached the hacknied quotation from Cicero, "Homines ad Deos nullâ re," &c., with propius converted into

*propriis.* Albinus is introduced by an epigraph from St. Augustine; and Sir Henry Halford is made to say, in the stiff language of Akenside,

"Me they sent,
To wait on pain," &c. &c.

Ruysch has a word or two from Horace: "Utilium sagax rerum." The immortal Haller is trumpeted on as "Artis Medicæ decus." Each of these last two mottoes would have done as well for the one as for the other great man, and each wants that which a motto should have—a distinct appropriateness. We turned with some curiosity to look for the motto given to Sir Anthony Carlisle, having some suspicion that he was his own biographer: here, however, we find no motto at all. But the absurdity is revived with Linacre, for whom Milton is quoted: "Nomen in exemplum sero servabimus ævo;" a regular smallhand copy. For Akenside, of course, something grand must be sought, and Virgil contributes "Quæ tibi quæ tali reddam pro carmine dona!" This is just as if Akenside's poem were of an order capable of making us forget his harsh, pompous, and unamiable conduct and manners as a physician. Sir Charles Clarke, we are sure, will smile at the fine conceit wherewith his life begins: "Rite maturos aperire partus." We hope that the mere difficulty of finding mottoes liable to no equivoques and puns will put a stop to this puerility, which savours little of the learning that refines and ennobles, and rather belongs to the frivolous literature that has flourished with those who have vainly considered themselves the Corinthian capitals of physic.

We subjoin Mr. Pettigrew's explanations:

"My object, I cannot but fear, has been imperfectly understood. I have not professed to write a regular history of medicine and surgery: my intention is, to give all the chief points of that history in a series of memoirs, taking for the subjects of these such individuals as have laboured most effectually in the advancement of any particular branch of medical science. But, as it would not be possible to obtain the BEST PORTRAITS, or materials, in the natural order of time, I have not felt myself bound to any specific method. The work is not consecutively paged; but each memoir is perfect in itself, and is separately numbered at the bottom of the page: the memoirs can therefore be arranged according to the fancy of the possessor of the work, either in the order of time or subject; or the LIVING can be separated altogether from the DEAD. In excuting such a work, I have conceived that I have been doing service to my profession. There exists no collection of medical portraits; there are many of the nobility, statesmen, divines, artists, &c. The portraits of medical men are scattered abroad, and there is some difficulty in obtaining information of the best: none but the BEST, however, shall be engraved, and as many originals as possible."

